# The pertinence of gastric cancer and interleukin 10–819 single nucleotide polymorphisms: a meta-analysis and systematic review

**DOI:** 10.1186/s12876-024-03151-9

**Published:** 2024-02-16

**Authors:** Qianqian Mao, Yanwen Liu, Xi Chen, Cheng Jiang Liu

**Affiliations:** 1 Medical School of Southeast University, 210000 Nanjing, China; 2 Department of Oncology, Zhongda Hospital, Medical School of Southeast University, 210000 Nanjing, China; 3 School of health, Brooks College (Sunnyvale) the United States of America, Department of epidemiology and statistics, School of public health, Medical College, Zhejiang University, 310000 Hangzhou, China; 4 Department of General Medicine, Affiliated Anqing First People’s Hospital of Anhui Medical University, 246000 Anqing, China

**Keywords:** IL-10 819, Gene, Single nucleotide polymorphism, Association, Gastric cancer

## Abstract

**Purpose:**

Cytokines regulate the interaction between the immune system and malignant tumors. Among them, interleukin-10 (IL-10) is a multifunctional anti-inflammatory cytokine mainly produced by immune cells. The correlation between gastric cancer and T/C single nucleotide polymorphism (SNP) of interleukin-10 (IL-10) promoter−819(rs1800871)was opaque and remained to be determined. We aim to explore the pertinence of gastric cancer and SNP of interleukin 10–819 by meta-analysis via five statistical models.

**Methods:**

Databases including PubMed, Cochrane Library, Embase, the Scopus, and Google Scholars were comprehensively retrieved for the eligible studies on the related topic from inception to March 2022. Odds ratios (ORs) were generated for dichotomous variants by meta-analysis in each model via STATA 17.0 MP. The statistical models comprised recessive model, over-dominant model, allele model, co-dominant model and dominant model. Subgroup analysis was performed to investigate the difference across races as well as the source of heterogeneity if necessary.

**Results:**

Eventually a total of 15 articles reporting 7779 patients were enrolled in our study. There were 2383 patients and 5396 controls, collectively. There was no correlation between gastric cancer and IL-10 819 in recessive model, co-dominant model or dominant model, and subgroup analysis showed that Asian, Latin American and Caucasian had no correlation with the risk of gastric cancer. In the allelic model, there was significant correlation between gastric cancer and IL-10 819 (OR = 3.96%, 95%CI: 3.28 to 3.78). In the over-dominant model, there is no correlation between gastric cancer and IL-10 819, but subgroup analysis uncovered significant vulnerability of Asian people with regard to gastric cancer.

**Conclusions:**

In our study, both Asians, Latin Americans, and Europeans showed an increased risk of gastric cancer in the allelic model, whereas only Asians showed significant susceptibility in the super dominant model. Of course, more large cohort studies are needed to confirm our results.

**Supplementary Information:**

The online version contains supplementary material available at 10.1186/s12876-024-03151-9.

## Introduction

Gastric cancer is the fifth most common cancer and the fourth leading cause of cancer death worldwide [1]. It is most common in Asia, Latin America and some European countries. In China, gastric cancer is the second most common cancer and the second leading cause of cancer-related death. Compared with most developed countries, China has higher mortality/morbidity and 5-year prevalence [1]. As an aggressive malignant tumor, GC has high incidence and poor prognosis. For a long time, identifying reliable biomarkers related to GC risk changes has been the research goal to improve the early detection of diseases [2]. 

Gastric cancer develops from multi-step gastropathy cascade, involving multiple causes, such as multiple gene sequence changes [3]. Activation of the proto-oncogene or the proliferating gene, inactivation, and mutation of the tumor suppressor gene can render cell growth uncontrolled. Single nucleotide polymorphism refers to the DNA sequence polymorphism caused by the mutation of a single nucleotide at the genome level due to the transformation, transversion and mutation of a single base [4]. With the deepening of human genomics research, many studies in recent ten years have shown that both genetic polymorphism and epigenetic changes are related to the risk of gastric cancer to some extent [5]. 

Cytokines are small molecular peptides or glycoproteins that are synthesized and secreted by a variety of tissue cells. Interleukins, interferons, tumor necrosis factors, hematopoietic factors, growth factors, and chemokines are collectively referred to as cytokines. Cytokines are involved in the regulation of almost all types of cellular responses, such as immune proliferation, differentiation and effector functions, and are essential for immune cells to fight against tumor cells and pathogens [6]. Cytokines may have pro-inflammatory or anti-inflammatory activities, and may be involved in immunomodulatory activities according to microenvironment [7]. Previous studies have shown that IL-10 can increase proliferation, accelerate cell growth and prolong cell survival of melanoma cells [8]. 

Compared with the previous articles, we have included more articles, which means that we have included a wider range of people, better extrapolating and better distinguishing regions and races. At the same time, we have enlarged the sample size. From the statistical point of view, this led to the reduction of random error and confidence interval, so the data became more stable and convincing. From the model point of view, the previous research did not involve so many genetic models, which may lead to the lack of related genetic models, and our advantage lies in the profound interpretation from the perspective of genetics and clinic [9–12]. 

Therefore, we conducted this meta-analysis to further update the evidence of evidence-based medicine, deeply clarify the role of IL-10 819 gene polymorphism in gastric cancer, provide reference for clinical work, and better evaluate the potential correlation between IL-10 819 gene polymorphism and gastric cancer risk.

## Method

### Search strategy

Databases including PubMed, Cochrane Library, Embase, the Scopus, and Google Scholars were comprehensively retrieved for the eligible studies on the related topic from inception to March 2022 with the following Mesh terms:(“Interleukin10“ OR ”IL-10“ OR ”Interleukin“ OR ”Cytokine“) AND (”Gene“ OR ”Polymorphism“ OR ”Variant“ OR ”SNP“) AND (“Neoplasm, Stomach” OR “Stomach Neoplasm” OR “Neoplasms, Stomach” OR “Gastric Neoplasms” OR “Gastric Neoplasm” OR “Neoplasm, Gastric” OR “Neoplasms, Gastric” OR “Cancer of Stomach” OR “Stomach Cancers” OR “Gastric Cancer” OR “Cancer, Gastric” OR “Cancers, Gastric” OR “Gastric Cancers” OR “Stomach Cancer” OR “Cancer, Stomach” OR “Cancers, Stomach” OR “Cancer of the Stomach” OR “Gastric Cancer, Familial Diffuse”).

### Criteria for inclusion and exclusion

Inclusion criteria:

1. Any study describing the association of the IL-10-819 C/T SNP with gastric cancer;
2. The number of controls and gastric cancer cases reported in any study;
3. Any study reporting the number of individuals of each genotype (TT, TC, CC) in cases and controls;
4. Results are expressed as odds ratios (ORs) with 95% confidence intervals (CIs);
5. The study is a case-control or nested case-control study.

Exclusion criteria:

1. There is no control group in the literature;
2. In the literature trial, the design is not rigorous (such as the diagnostic and efficacy judgment standard are not standardized, the sample data are unclear or incomplete, etcetera);
3. The statistical approach is inadequate;
4. Repeated publications, review and conference papers and so on.

### Literature screening and data extraction

Two investigators independently searched, assessed, and extracted data from literature. Any discrepancy was arbitrated by a senior investigator. Data were collected including first author, year of publication, country, ethnic origin (classified as Asian, Caucasian or mestizo), genotyping method, number of cases and controls.

### Quality evaluation literature screening

The NOS scale was adopted as the evaluation method. The evaluation content includes three aspects: (1) selection of case and control groups; (2) comparability between the case and controls; (3) exposure of the case group and control group. The score is 1–9 points, 1–5 points are low-quality, 6–9 points are high-quality, and low-quality literature is directly discarded. If there are differences between researchers, a third-party ruling may be used.

### Statistical analysis

The software utilized was STATA version 17.0 MP. Mean difference (MD) was generated as effect-size for continuous variants. Odds ratios (ORs) were generated for dichotomous variants. If I^2^ ≤ 50% and *P* > 0.01, a fixed effect model would be implemented, otherwise a random effect model would be performed. If I^2^ > 75%, then subgroup analysis would be performed to explore the source of heterogeneity. Publication bias was assessed by Egger’s test. Probability value *P* < 0.05 was considered statistically significant.

## Result

### Literature search and study selection

**Fig. 1:**
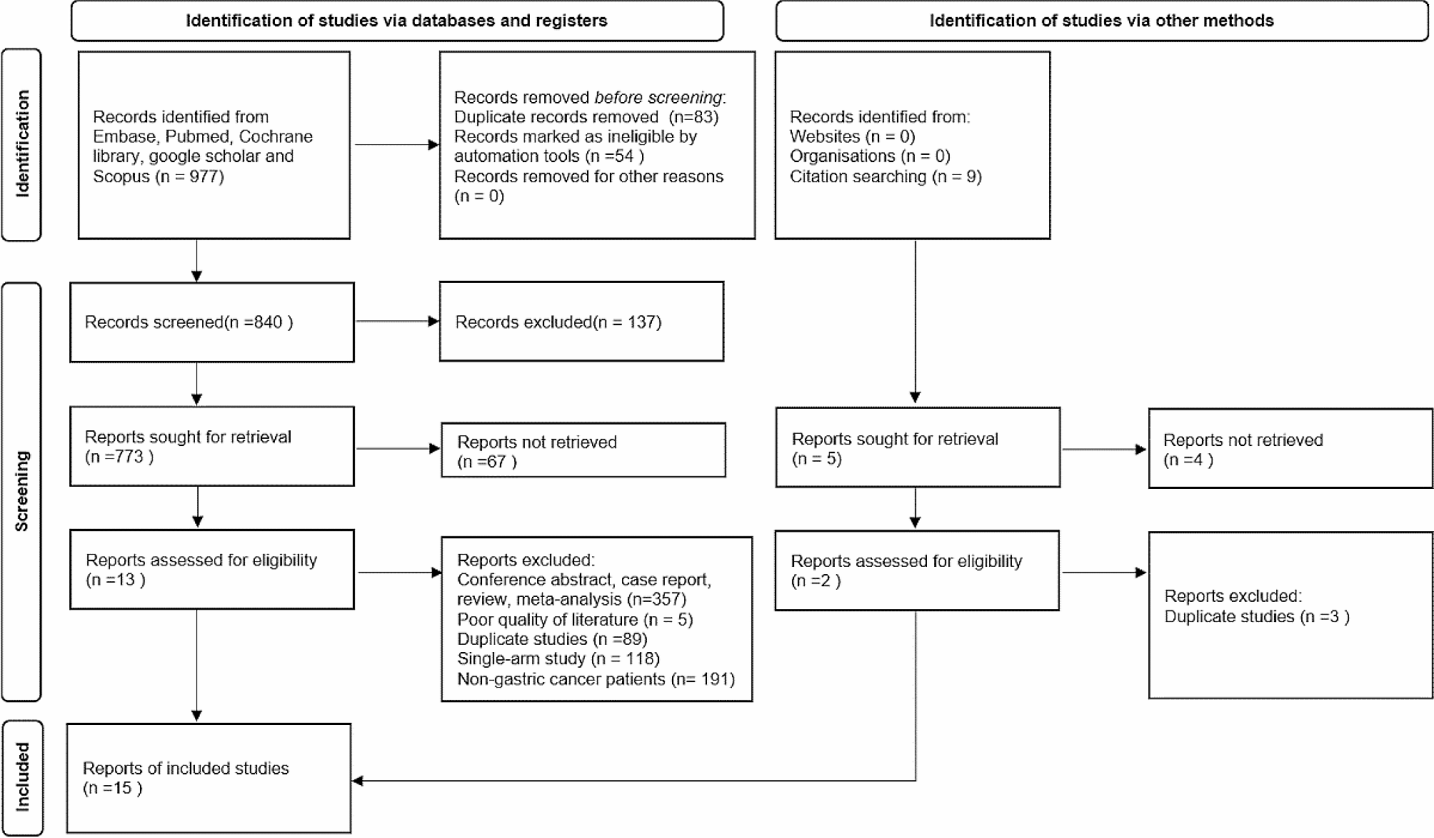
Flow chart of article inclusion and exclusion

A total of 986 potentially relevant articles were retrieved. After screening, a total of 15 articles meeting the inclusion criteria of Meta-analysis were included (Fig. 1) [13–27]. All eligible studies were published between 2003 and 2018. Thirteen of them were published in English and two in Chinese. These 15 articles had high NOS quality scores, with ratings fluctuating between 6 and 8. A total of 7,778 people were involved in 15 studies, including 2,383 cases and 5,395 controls. The basic characteristics of the included studies are shown in Table 1. Genes are divided into wild-type and mutant. Gene mutation is the transformation of wild-type genes into mutant genes. In this paper, the wild-type gene of IL-10 819 is denoted by A, and the mutant gene is denoted by B.

**Table 1:** The features of the included study

First author	Year of	Genotyping method	Total sample size	Number of controls	Number of cases	Study	Ethnic	NOS
Wu MS	2003	Direct Sequencing	450	230	220	China	Asians	7
Savage SA	2004	ABI Genetic Analyzer	466	382	84	China	Asians	5
Alpízar-Alpízar W	2005	Pyrosequencing	90	45	45	Costa Rica	Latinos	6
Zambon CF	2005	TaqMan	773	644	129	Italy	Caucasians	5
Kamangar F	2006	TaqMan	250	152	98	Finland	Caucasians	8
Sugimoto M	2007	ASP	273	168	105	Iapan	Asians	6.5
Crusius JB	2008	ABI real-time PCR	1323	1094	229	European	Caucasians	8.5
Xiao H	2009	RFLP	844	624	220	China	Asians	6
Ko KP	2009	SNaPshot	409	326	83	Korea	Asians	7
Su SP	2010	RFLP	143	100	43	China	Asians	6.5
Liu J	2011	RFLP	477	243	234	China	Asians	6.5
Zeng X	2012	ABI Prism SNaPshot Multiplex kit	305	154	151	China	Asians	6
Li L	2016	RFLP	405	248	157	China	Asians	6
Sarah Yang	2017	Affymetrix Axiom Exom 319 Array	1131	754	377	Korea	Asians	7
Liu Sa	2018	RFLP	440	232	208	China	Asians	7

### Statistical analysis results

Significant associations with the risk of gastric cancer were detected for the IL-10 819.

**Fig. 2:**
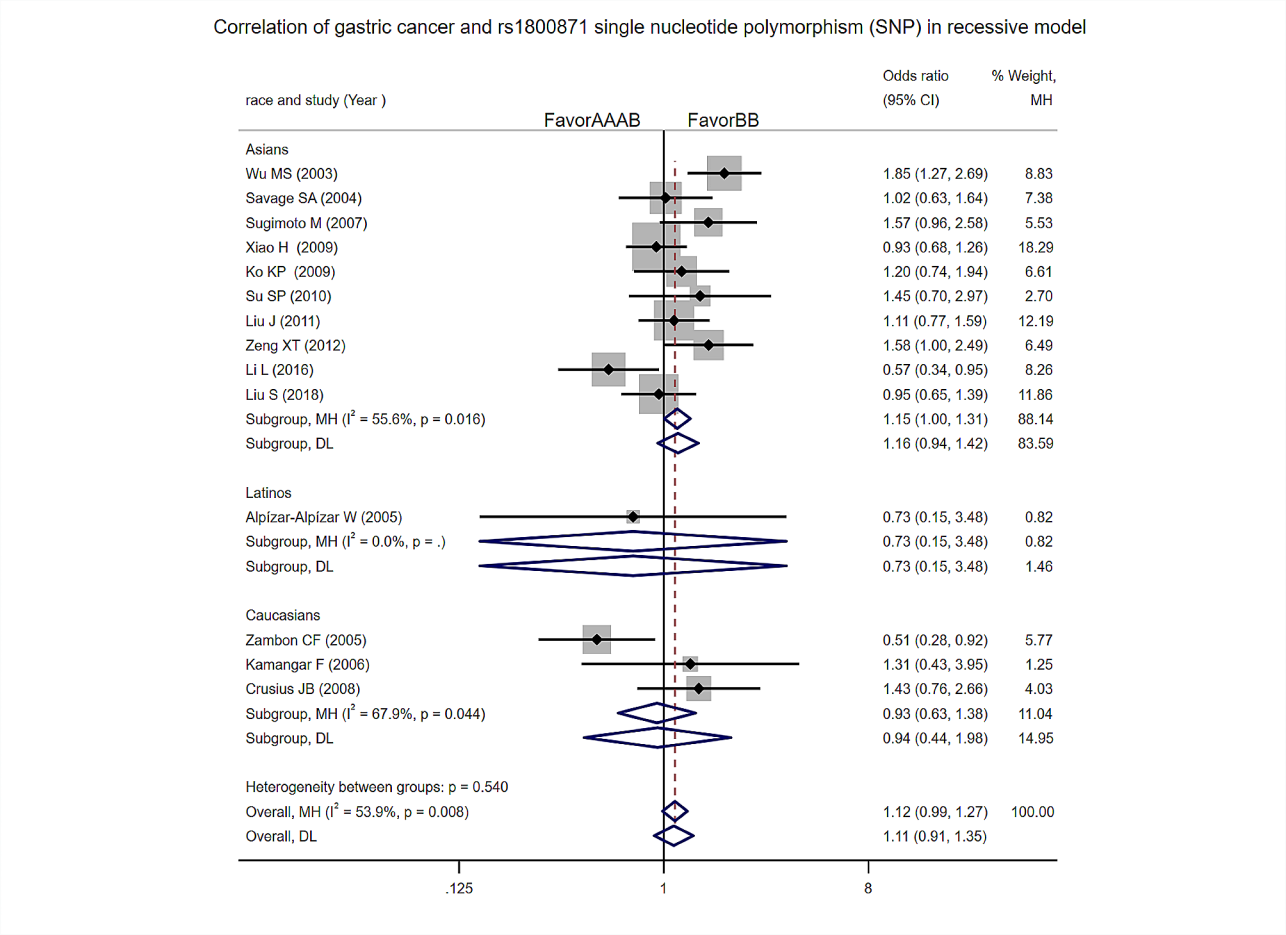
Recessive model

Recessive model: Heterogeneity test analysis: The study showed statistically significant heterogeneity, the Meta-analysis results showed that the two groups were significantly different. The difference between the two groups was not statistically significant [OR = 1.11, 95%CI (0.91, 1.35), *p* = 0.315]. Subgroup analysis by ethnicity found no difference in the risk of gastric cancer among Asians, Latinos, and Caucasians in this model (Fig. 2).

**Fig. 3:**
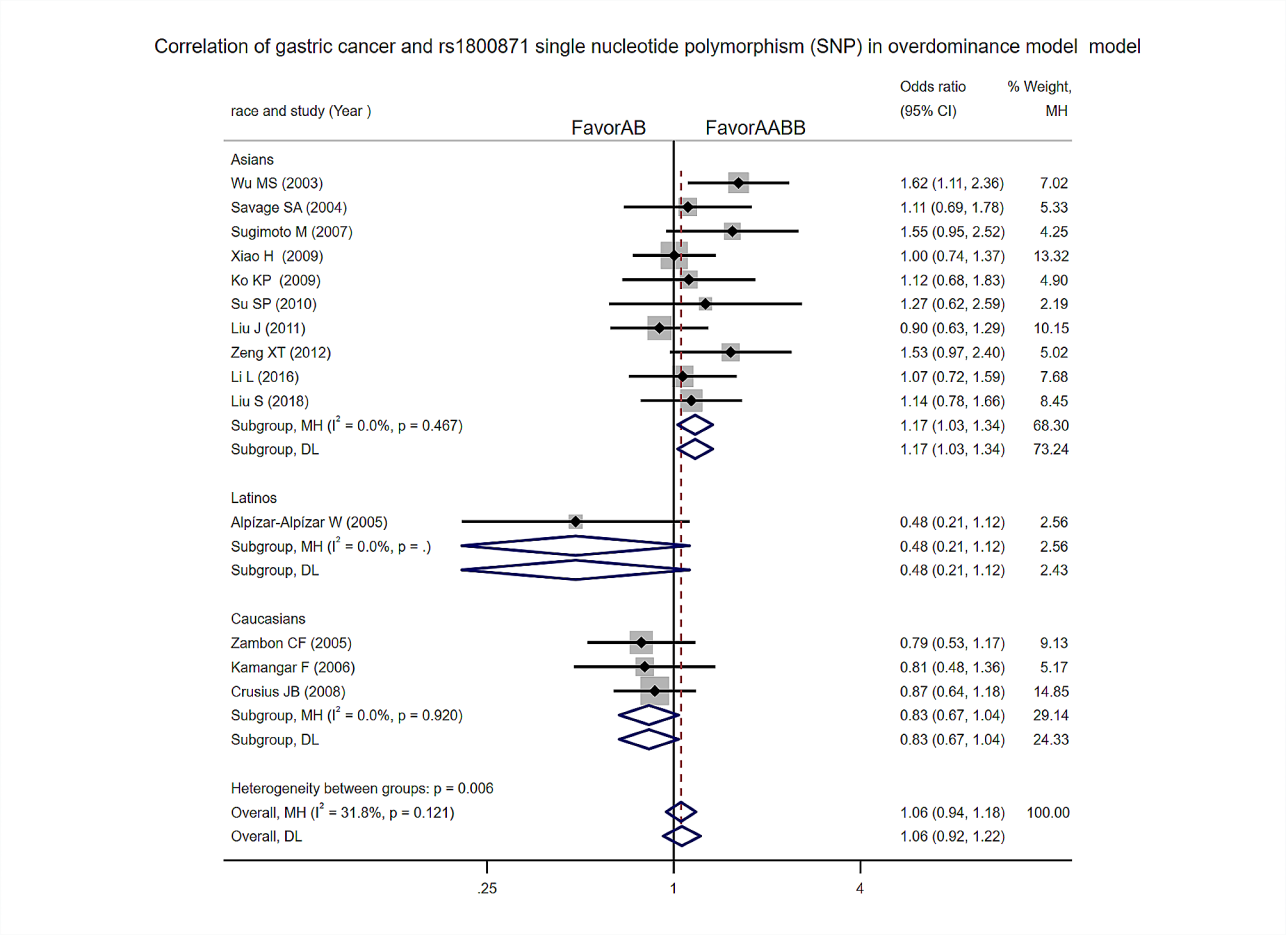
Super-dominant model

Super-dominant model: Heterogeneity test analysis: This research shows statistically insignificant heterogeneity. The difference was not statistically significant [OR = 1.06, 95%CI (0.94, 1.18), *p* = 0.341]. A Subgroup analysis based on ethnicity found significant statistical heterogeneity in Asian related studies (I^2^ = 0%, *P* = 0.467). The meta-analysis showed significant differences between the two groups [OR = 1.17, 95%CI (1.03, 1.34), *p* = 0.017] (Fig. 3). The risk of leukemia in the AA + BB genotype population is higher than that in the AB genotype population [OR = 1.43, 95%CI (1.06, 1.91), *P* = 0.02], this difference only exists in the Asian population [OR = 2.00, 95%CI (1.37, 2.92), *P* = 0.0003], no significant difference among Caucasians [OR = 1.13, 95%CI (0.86, 1.49), *P* = 0.37].

**Fig. 4:**
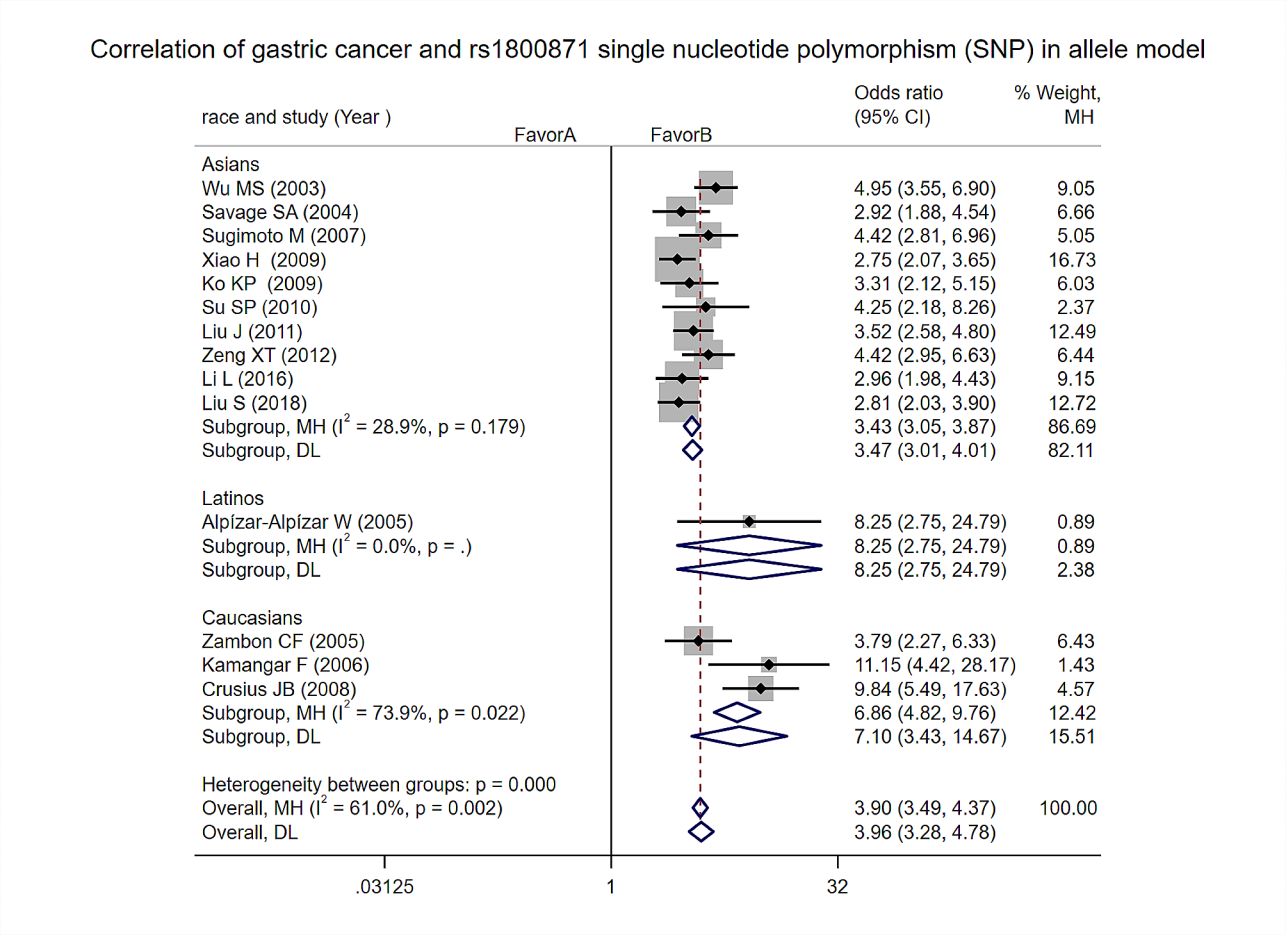
Allelic model

Allelic model: Heterogeneity test analysis: There was significant statistical heterogeneity in the study, and the meta-analysis results showed that the difference between the two groups Statistically significant [OR = 3.96, 95%CI (3.28, 3.78), *p* = 0], indicating that the IL-10–819 C > T variant genotype significantly increased the risk in the allelic model. Subgroup analysis by ethnicity found that the IL-10–819 C > T variant genotype significantly increased risk in Asians, Latinos, and Caucasians (Fig. 4).

#### Codominant models

**Fig. 5:**
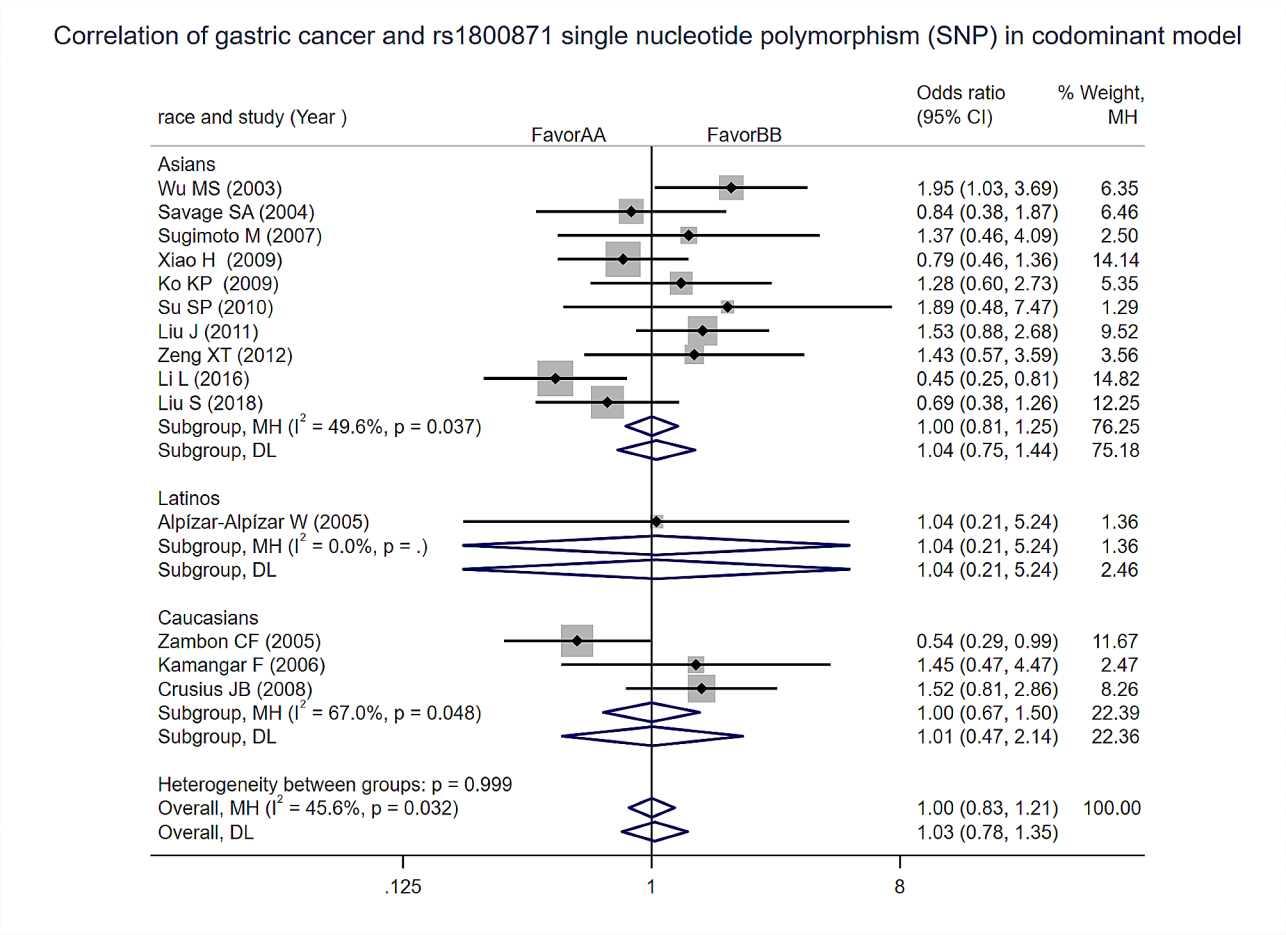
Homozygous ofCodominant models

Homozygous: Heterogeneity test analysis: There was no significant statistical heterogeneity in the study, the Meta-analysis results showed that the difference between the two groups was not statistically significant [OR = 1, 95% CI (0.83, 1.21), *p* = 0.999]. Subgroup analysis by ethnicity found no difference in the risk of gastric cancer among Asians, Latinos, and Caucasians in this model (Fig. 5).

**Fig. 6:**
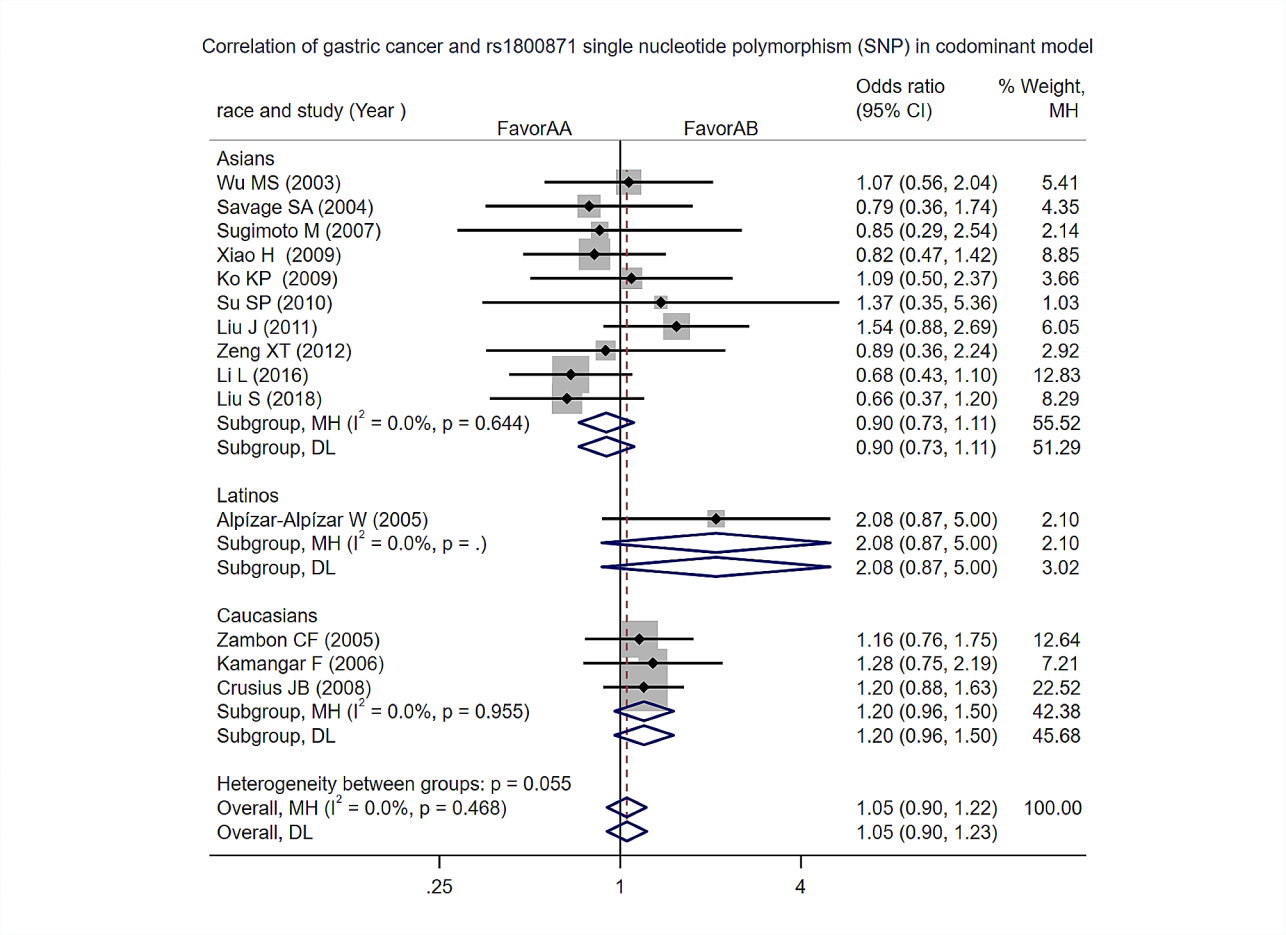
Heterozygote of Codominant models

Heterozygote: Heterogeneity test analysis: There was no significant statistical heterogeneity in the study, and the Meta-analysis results showed that the difference between the two groups was not statistically significant [OR = 1.05, 95% CI (0.9, 1.22), *p* = 0.055]. Subgroup analysis by ethnicity found no difference in the risk of gastric cancer among Asians, Latinos, and Caucasians in this model (Fig. 6).

**Fig. 7:**
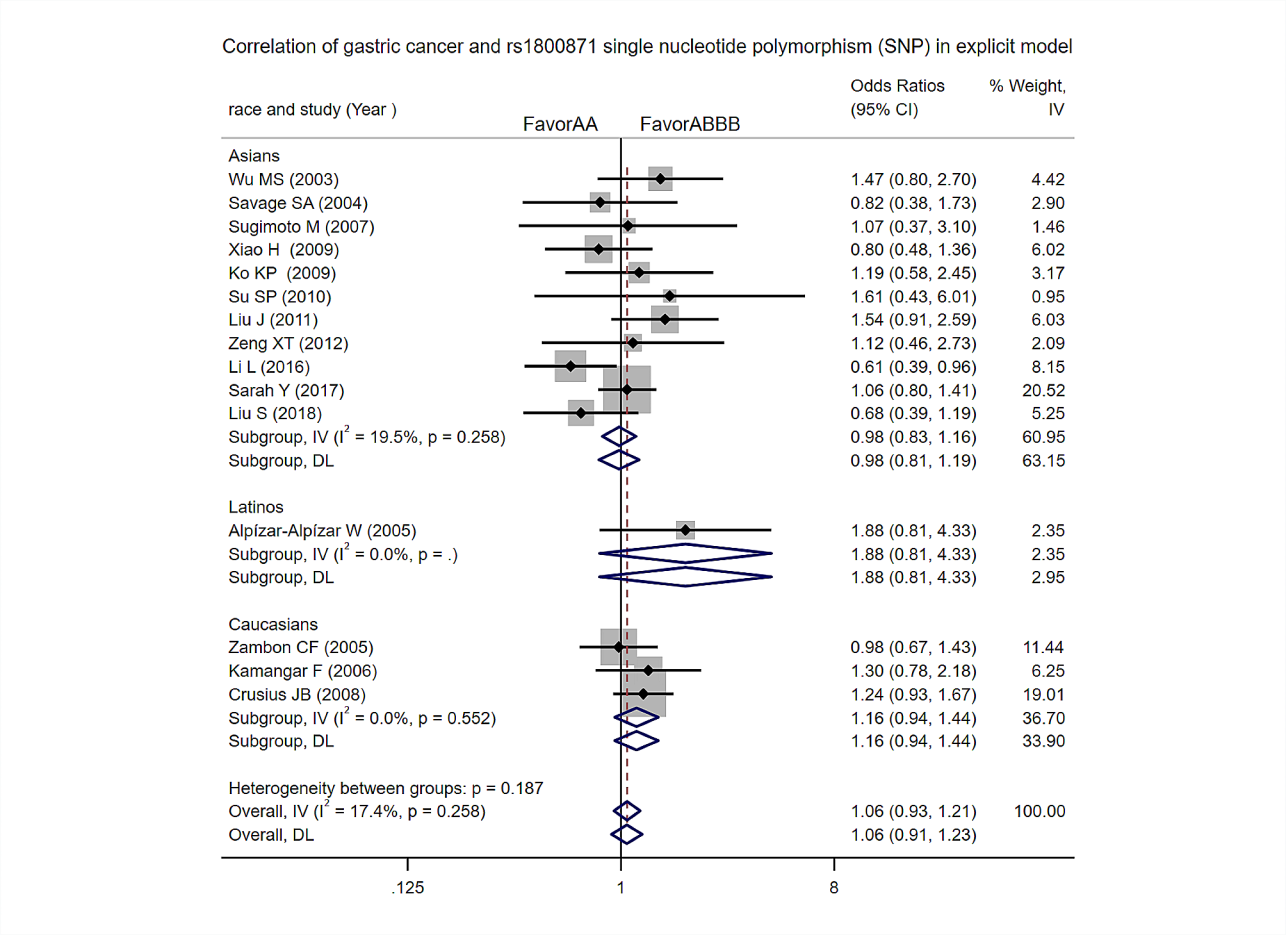
Dominant model

Dominant model: Heterogeneity test analysis: There was no significant statistical heterogeneity in the study. The difference was not statistically significant [OR = 1.06, 95%CI (0.93, 1.21), *p* = 0.999]. Subgroup analyses by ethnicity revealed that there was no difference in gastric cancer risk among Asians, Latinos and Caucasians in this model (Fig. 7).

#### Publication bias analysis

Egger’s test showed no significant Publication bias in all models(*P* > 0.05).

## Discussion

In recent years, many studies have attempted to link IL-10 gene polymorphisms with gastric cancer risk, but these studies have yielded conflicting results. For example, Sarah, Jie Liu, Su SPbelieve that IL-10 819 gene polymorphism is not related to the risk of gastric cancer [22, 23, 26]. 

L. Li et al. showed that IL-10 C819T polymorphism was associated with increased risk of gastric cancer in co-dominant, dominant and recessive models [25]. Xiangting Zeng et al. think that the existence of IL-10-819 C allele is related to the increased risk of gastric cancer development [24]. The above different results may be related to the lack of proper models and the heterogeneity of the studied population. Therefore, we performed this meta-analysis to better analyze the correlation between IL-10 gene polymorphisms and gastric cancer.

Previous studies have shown that excessive and sustained production of pro-inflammatory mediators is a major factor in tumor promotion and progression [28]. Interleukins are a group of strong immunomodulatory cytokines secreted by lymphocytes, antigen-presenting cells and endothelial cells and are essential for the maintenance of normal immune function [20]. Any alteration in the level of interleukin expression or disruption of its action may result in severe immune dysfunction and lead to the development of malignancy [21]. Therefore, it is biologically plausible that functional interleukin gene polymorphisms may be associated with the risk of developing gastric cancer.IL-10 is a multifunctional cytokine with immunosuppressive and antiangiogenic functions, and it reduces the expression of MHC class in antigen presenting cells (APCs)17 and tumor cells by downregulating the expression of MHC class or inhibit antigen presentation, thereby contributing to an immunosuppressive environment [29–31]. IL-10 is located on chromosome 1 of 1q31-32 with a span of approximately 4.7 kb, including 4 introns and 5 exons. SNPs in the promoter region of the IL10 gene have been shown to alter IL-10 mRNA and protein levels [8]. Individual genetic susceptibility diversity is established based on genetic polymorphisms, and sequence variation in the gene promoter of cytokines may alter the associated transcription factor recognition sites, while the binding of specific recognition sequences in the promoter to regulatory factors affects the transcriptional level of gene expression, thus influencing transcriptional activation and cytokine production. For example, polymorphisms on the IL-10 promoter site-1082 are distributed at recognition sites similar to ETS and thus may have an effect on the binding of this transcription factor, which has been shown to act as a negative regulator in IL-2 production.

Our meta-analysis involved 15 studies, including 2383 cases and 5395 controls. The results revealed that there was no significant relationship between promoter polymorphism of IL10 819 and GC risk of recessive, super dominant, co-dominant and dominant genes models. However, compared with IL-10 819 wild type allele, IL-10 819 mutant allele can increase the risk of GC, which indicates that the mutant allele of IL-10,819 C/T polymorphism is dangerous. In other words, in the allele model, there is a significant correlation between gastric cancer and IL-10 819. In recent years, a number of studies have shown that the presence of the IL-10 819 C allele is associated with an increased risk of gastric cancer [13, 18], and further studies have found that the IL-10 819 C allele is associated with an increased risk of gastric cancer in patients with HP infection [24]. Zambon pointed out that the IL-10-819 TT genotype is related to intestinal metaplasia and NCGC [16]. The genotyping detection of anti-inflammatory cytokines will help to detect individuals at higher risk of gastric cancer.

Heterogeneity is a potential problem in meta-analysis. The data will be affected by subgroup differences, so subgroup analysis is needed. Most of the published related studies have chosen Helicobacter pylori for subgroup analysis, and our research has chosen race for analysis. Subgroup analysis showed that in the allele genetic model of IL-10 819 C/T polymorphism, the mutant allele was significantly associated with the increased risk of gastric cancer among Asians, Latinos and Caucasians. In addition, in the super dominant model, there is no correlation between gastric cancer and IL-10 819, but subgroup analysis shows that Homozygous genotype is related to the significantly increased risk of Asians. We found that overall, heterogeneity between studies was greatly reduced after data stratification by subject race, indicating that heterogeneity between included studies could be partially explained by race-induced differences. According to the above conclusions, we can consider whether the difference of race will affect the immunotherapy of gastric cancer from the perspective of genes, and then we can screen out the corresponding dominant population to achieve accurate treatment.

Changes in human DNA sequences can influence the occurrence and progression of human diseases. Single nucleotide polymorphism (SNP) is also key to individualized medical treatment. This study suggests that IL-10 819 gene polymorphism may be a genetic biomarker of gastric cancer, and clinical detection of gene mutation typing can provide scientific theoretical basis to further reveal the biological mechanism of the prognosis of gastric cancer.

In addition, in the future, we can investigate the role of IL-10 819 single nucleotide polymorphisms on in the prognosis of gastric cancer at different stages more accurately by further elaborate studies with cancer gene mutation screening kits.

The limitations of our meta-analysis should be considered when interpreting the results. First of all, the number of articles included in this paper is limited, and the sample size of our research is small. Secondly, the races involved are limited (only Asian, Hispanic and white). Thirdly, this paper only uses recessive model, super dominant model, allele model, co-dominant model and dominant model for analysis. More models are expected to explore the relationship between IL-10 819 promoter polymorphism and gastric cancer.

## Conclusion

Generally speaking, our meta-analysis study in different populations confirmed that interleukin-10-819 promoter polymorphism could be used as a genetic biomarker of gastric cancer. If they have the financial ability, it is suggested that people with family history of gastric cancer should be tested for susceptibility genes for early detection and treatment. In addition, our findings still need further well-designed research to confirm, and the possible role of other interleukin gene polymorphisms in gastric cancer also needs further exploration.

### Electronic supplementary material

Below is the link to the electronic supplementary material.

Supplementary Material 1

Supplementary Material 2

## Data Availability

The datasets used and/or analysed during the current study available from the corresponding author on reasonable request.
