# Supplementary material for: The pertinence of gastric cancer and interleukin 10–819 single nucleotide polymorphisms: a meta-analysis and systematic review
Source: BMC Gastroenterol. 2024 Feb 16;24:76. doi: 10.1186/s12876-024-03151-9 (PMC10874039; doi:10.1186/s12876-024-03151-9)
Supplement: Supplementary file 2 — Supplementary Material 2 [file 12876_2024_3151_MOESM2_ESM.docx]

Supplementary Table 1. Number of genotypes in gastric cancer cases and controls in the included.

| study | caseAA | caseAB | caseBB | controlAA | controlAB | controlBB |
| --- | --- | --- | --- | --- | --- | --- |
| Wu MS | 27 | 105 | 88 | 20 | 83 | 127 |
| Savage SA | 9 | 38 | 37 | 49 | 163 | 170 |
| Alpízar-Alpízar W | 25 | 16 | 4 | 18 | 24 | 3 |
| Zambon CF | 70 | 42 | 17 | 353 | 245 | 46 |
| Kamangar F | 58 | 35 | 5 | 80 | 62 | 10 |
| Sugimoto M | 6 | 57 | 42 | 9 | 73 | 86 |
| Crusius JB | 145 | 72 | 12 | 636 | 378 | 80 |
| Xiao H | 20 | 100 | 100 | 69 | 283 | 272 |
| Ko KP | 11 | 33 | 39 | 37 | 121 | 168 |
| Su SP | 4 | 21 | 18 | 6 | 43 | 51 |
| Liu J | 39 | 96 | 99 | 28 | 106 | 109 |
| Zeng X | 11 | 80 | 60 | 10 | 65 | 78 |
| Li L | 38 | 83 | 36 | 85 | 127 | 36 |
| Sarah Yang | 173 | 204 | / | 368 | 386 | / |
| Liu Sa | 23 | 100 | 85 | 36 | 104 | 92 |
